# A mutated *dph3* gene causes sensitivity of *Schizosaccharomyces pombe* cells to cytotoxic agents

**DOI:** 10.1007/s00294-017-0711-x

**Published:** 2017-05-29

**Authors:** Desirée Villahermosa, Karen Knapp, Oliver Fleck

**Affiliations:** 1 North West Cancer Research Institute, Bangor University, Bangor, LL57 2UW UK; 2 Present Address: Department of Pathology, Dunedin School of Medicine, University of Otago, Dunedin, 9054 New Zealand

**Keywords:** DNA damage response, Elongator, Genome stability, Sordarin, Transcriptional interference, Translation elongation factor eEF2, tRNA modifications

## Abstract

Dph3 is involved in diphthamide modification of the eukaryotic translation elongation factor eEF2 and in Elongator-mediated modifications of tRNAs, where a 5-methoxycarbonyl-methyl moiety is added to wobble uridines. Lack of such modifications affects protein synthesis due to inaccurate translation of mRNAs at ribosomes. We have discovered that integration of markers at the *msh3* locus of *Schizosaccharomyces pombe* impaired the function of the nearby located *dph3* gene. Such integrations rendered cells sensitive to the cytotoxic drugs hydroxyurea and methyl methanesulfonate. We constructed *dph3* and *msh3* strains with mutated ATG start codons (*ATGmut*), which allowed investigating drug sensitivity without potential interference by marker insertions. The *dph3*-*ATGmut* and a *dph3::loxP*-*ura4*-*loxM* gene disruption strain, but not *msh3*-*ATGmut*, turned out to be sensitive to hydroxyurea and methyl methanesulfonate, likewise the strains with cassettes integrated at the *msh3* locus. The fungicide sordarin, which inhibits diphthamide modified eEF2 of *Saccharomyces cerevisiae*, barely affected survival of wild type and *msh3Δ S. pombe* cells, while the *dph3Δ* mutant was sensitive. The *msh3*-*ATG* mutation, but not *dph3Δ* or the *dph3*-*ATG* mutation caused a defect in mating-type switching, indicating that the *ura4* marker at the *dph3* locus did not interfere with Msh3 function. We conclude that Dph3 is required for cellular resistance to the fungicide sordarin and to the cytotoxic drugs hydroxyurea and methyl methanesulfonate. This is likely mediated by efficient translation of proteins in response to DNA damage and replication stress.

**Electronic supplementary material:**

The online version of this article (doi:10.1007/s00294-017-0711-x) contains supplementary material, which is available to authorized users.

## Introduction

Dph3 is involved in the Dph pathway for a unique diphthamide modification of eukaryotic translation elongation factor 2 (eEF2) (Schaffrath et al. 2014). Modified eEF2 ensures correct movement of tRNAs and mRNAs along ribosomes and allows rapid addition of amino acids to growing peptide chains during translation. Diphthamide modification by Dph1–Dph7 is best understood in *Saccharomyces cerevisiae* (Schaffrath et al. 2014). Dph3 provides electrons to Dph1–Dph2 for the catalysis of 3-amino-carboxypropyl on a specific histidine of eEF2 (Liu et al. 2004; Dong et al. 2014). Subsequently, Dph5 tetra-methylates the 3-amino-carboxypropyl-histidine, which is then converted to diphthine by Dph7. Finally, Dph6 modifies diphthine to diphthamide (Lin et al. 2014; Schaffrath et al. 2014). Diphthamide modified eEF2 is a target of bacterial toxins, such as diphtheria toxin, and fungicides such as sordarin (Van Ness et al. 1980; Chen et al. 1985; Domínguez and Martin 1998; Jablonowski and Schaffrath 2007). Diphtheria toxin is an ADP ribosylase of *Corynebacterium diphtheria* that ADP-ribosylates the diphthamide modification of eEF2; thereby inhibiting its function, which ultimately leads to cell death and causes diphtheria in humans (Schaffrath et al. 2014). Sordarin is a metabolite produced by the fungus *Sordaria araneosa* and inhibits diphthamide modified eEF2 in *S. cerevisiae* through a sordarin specificity region located N-terminal to the diphthamide (Shastry et al. 2001). Inhibition by sordarin occurs by blocking ribosomal translocation through stalling of eEF2, thereby preventing translation of mRNAs (Justice et al. 1998; Domínguez et al. 1999). *S. cerevisiae* and some other fungi are sensitive to sordarin, whereas deletions of any of the *DPH* genes confer resistance (Domínguez et al. 1999; Shastry et al. 2001; Bär et al. 2008; Uthman et al. 2013).

Dph3 also plays a role in the first step of tRNA wobble uridine modifications carried out by the Elongator complex to form 5-carbonylmethyl-uridine (cm^5^U_34_) (Huang et al. 2005; Bär et al. 2008; Greenwood et al. 2009). The cm^5^U_34_ modification is methylated to 5-methoxycarbonyl-methyl-uridine (mcm^5^U_34_) by Trm9–Trm112 and to 5-carbamoylmethyl-uridine (ncm^5^U_34_) by an unknown enzymatic activity (Karlsborn et al. 2014; Deng et al. 2015). The mcm^5^ modified uridine can be further thiolated to mcm^5^s^2^ by the Urm1 pathway (Nakai et al. 2008; Leidel et al. 2009). Both, tRNA modifications and diphthamide-modified eEF2 ensure optimal translation of mRNAs to proteins (Svejstrup 2007; Schaffrath et al. 2014; Gu et al. 2014; Nedialkova and Leidel 2015; Thiaville and de Crécy-Lagard 2015).

In this study, we discovered that replacement of the open reading frame of *S. pombe msh3* by gene disruption cassettes interfered with *dph3* functions, which rendered cells sensitive to hydroxyurea (HU) and methyl methanesulfonate (MMS). Msh3 is a eukaryotic homologue of bacterial MutS. MutS is a DNA mismatch binding protein that initiates removal of mismatched and unpaired nucleotides, which were incorporated into the nascent strand during replication (Marti et al. 2002; Jiricny 2013). We verified with *dph3* and *msh3* strains with mutated ATG start codons (*ATGmut*) that drug sensitivity was indeed due to an impaired *dph3* function. Thus, Dph3 plays a role in response to DNA damage and replication stress, likely through modifications of tRNA and/or eEF2, which allow efficient biosynthesis of DNA damage response proteins.

## Results

### Gene disruptions of *msh3* caused HU and MMS sensitivity by interference with functions of the flanking *dph3* gene

**Fig. 1:**
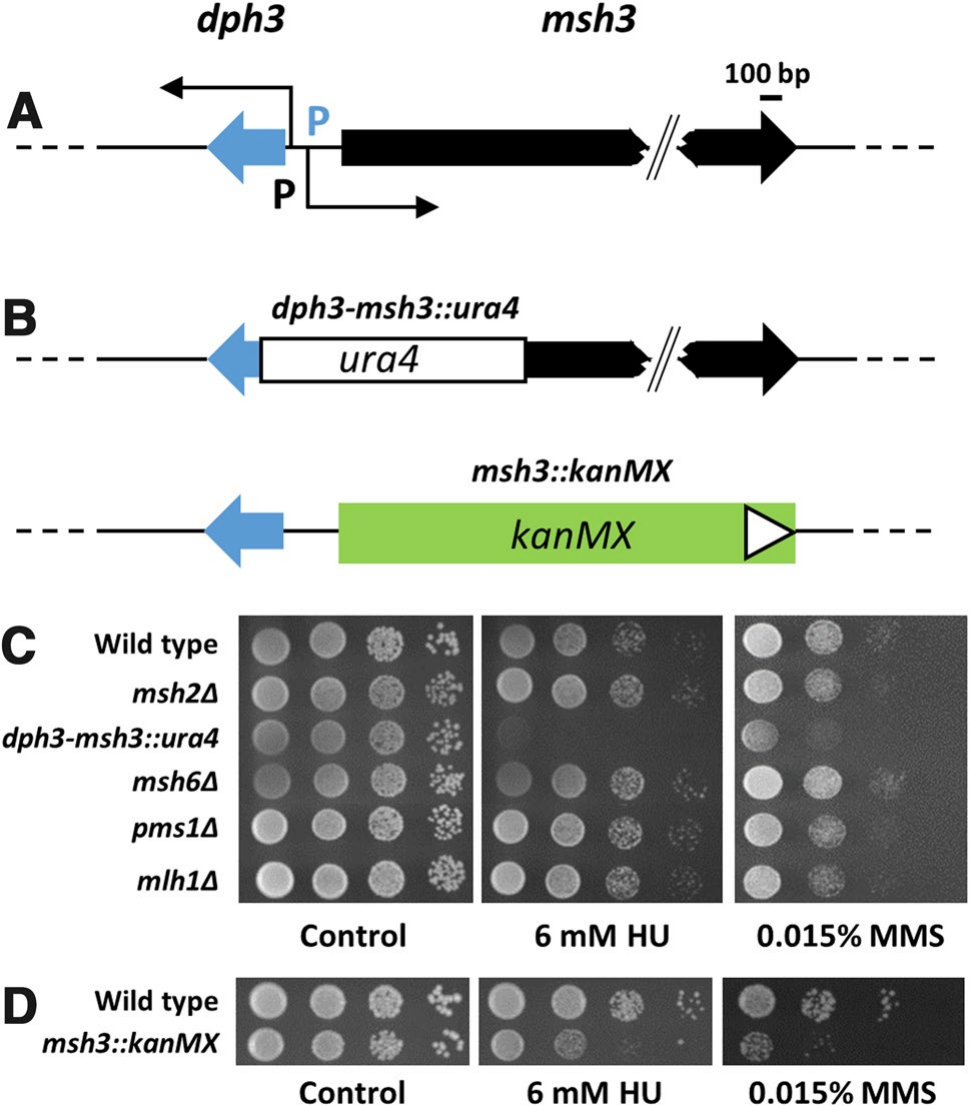
Test for drug sensitivity of MMR mutants. **a** Structural arrangement of the *dph3* and *msh3* genes. P’s designate promoters of *dph3* and *msh3*. *Bent arrows* indicate the transcription start sites according to Li et al. (2015). **b** Schematic of the *dph3*–*msh3::ura4* and *msh3::kanMX* disruptions. The *white triangle* indicates the orientation of the *kanMX* cassette, pointing towards the 3′ end. The orientation of the *ura4* gene in *dph3*–*msh3::ura4* is unknown. **c** The *dph3*–*msh3::ura4* strain, in which *ura4* replaced parts of the *dph3* and *msh3* genes, was sensitive to HU and MMS. All other MMR mutants were not sensitive when compared to wild type. **d** An *msh3::kanMX* strain was sensitive to HU and MMS

The *S. pombe msh3* and *dph3* genes share an intergenic region of only 268 base pairs (bp) between the two ATG start codons (Wood et al. 2002; http://www.pombase.org/). Data obtained by functional genomics further revealed that the intergenic region constitutes the 5′ untranslated regions (5′ UTR) of the *msh3* and *dph3* mRNAs, with divergent and likely overlapping promoters (Li et al. 2015) (Fig. 1a). Our initial work aimed to analyse functions of *msh3* in genome stability. The original *msh3* disruption was constructed before the sequence of the gene was determined (Fleck et al. 1992), thereby deleting parts of both *dph3* and *msh3*; hence termed *dph3*–*msh3::ura4* (or *dph3*–*msh3Δ*) from here on (Fig. 1b). Our previous data revealed that *S. pombe* Msh3 does not have a function in mismatch repair (MMR) of base–base mismatches and loops of one to four nucleotides (Tornier et al. 2001; Mansour et al. 2001; Marti et al. 2002; Villahermosa et al. 2017). To further understand the functions of MMR factors and particularly of Msh3, we examined sensitivity of *dph3*–*msh3::ura4* and the MMR mutants *msh2Δ*, *msh6Δ*, *pms1Δ* and *mlh1Δ* to HU and MMS. HU inhibits ribonucleotide reductase, thereby depleting the dNTP pools, which affects DNA replication and repair. MMS is an alkylating agent that causes DNA breaks. The *dph3*–*msh3::ura4* deletion strain, but none of the MMR mutants *msh2Δ*, *msh6Δ*, *pms1Δ* and *mlh1Δ* turned out to be sensitive to either drug (Fig. 1c).

**Fig. 2:**
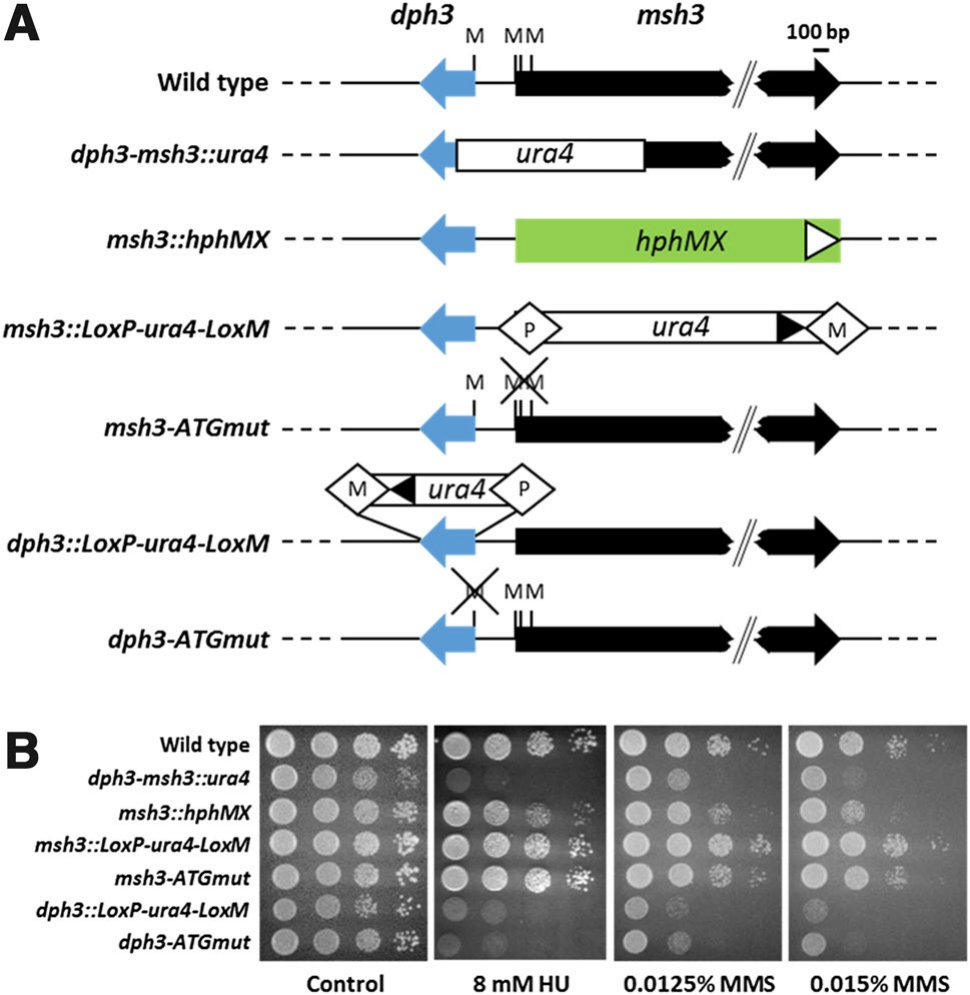
HU and MMS sensitivity is due to defective *dph3*. **a** Schematic of the genomic wild type loci and of gene disruptions and ATG mutants of *dph3* and *msh3*. The selection markers *ura4* and *hphMX* and the *lox* sites (white diamonds labelled with P and M) were not drawn to scale. M’s above the genes represent the start codons and, in the case of *msh3*, nearby ATG codons. *Crossed-out* M’s indicate that these codons have been mutated. *Triangles* indicate the orientation of the gene replacement cassettes pointing towards the 3′ end. **b** *dph3* mutants confer sensitivity to HU and MMS. Strains tested have the genotypic features illustrated in (**a**)

Because *ura4* in the *dph3*–*msh3::ura4* mutant disrupts two genes, we decided to construct a clean *msh3* deletion by replacing the entire open reading frame and no other parts with a *kanMX* cassette (Fig. 1b). The resulting *msh3::kanMX* deletion mutant was sensitive to HU and MMS like the original *dph3*–*msh3::ura4* mutant (Fig. 1d). However, when we replaced the open reading frame of *msh3* either with an *hphMX* cassette or with *ura4* flanked by *lox* sites (Fig. 2a), we noticed that drug sensitivity was not necessarily due to loss of Msh3. The *msh3::hphMX* mutant showed some sensitivity to MMS, but barely to HU, whereas the *msh3::loxP*-*ura4*-*loxM* was not sensitive to either drug (Fig. 2b). The observed effects on drug sensitivity can be explained by the presence of mutations in the intergenic region and by positional effects of the inserted markers, which interfere with *dph3* transcription. Sequencing of the 5′ UTR of the *msh3::kanMX* and *msh3::hphMX* strains revealed the wild-type sequence, ruling out the first possibility.

### A *dph3*-*ATGmut* strain, but not *msh3*-*ATGmut*, was sensitive to HU and MMS

We then decided to follow two approaches for testing whether drug sensitivity was caused by an impaired function of *dph3* or of *msh3*. We treated wild type, *dph3Δ* and *msh3Δ* with the fungicide sordarin and we constructed strains with mutated ATG start codons and tested them for sensitivity to HU and MMS. In the case of the *msh3*-*ATGmut* strain, we also mutated the fourth and the 31st codons, which both code for methionine (see also Fig. 2a). With the ATG mutations, we avoided that cassette integrations affected the promoters of the genes. The *dph3::loxP*-*ura4*-*loxM* and *dph3*-*ATGmut* strains, but not the *msh3*-*ATGmut* strain, were sensitive to HU and MMS (Fig. 2b). Thus, defective *dph3*, but not *msh3*, clearly caused drug sensitivity. Furthermore, it became evident that integration of *kanMX* or *hphMX* at the *msh3* locus interfered with *dph3* function.

### *S. pombe* Dph3 confers resistance to sordarin

**Fig. 3:**
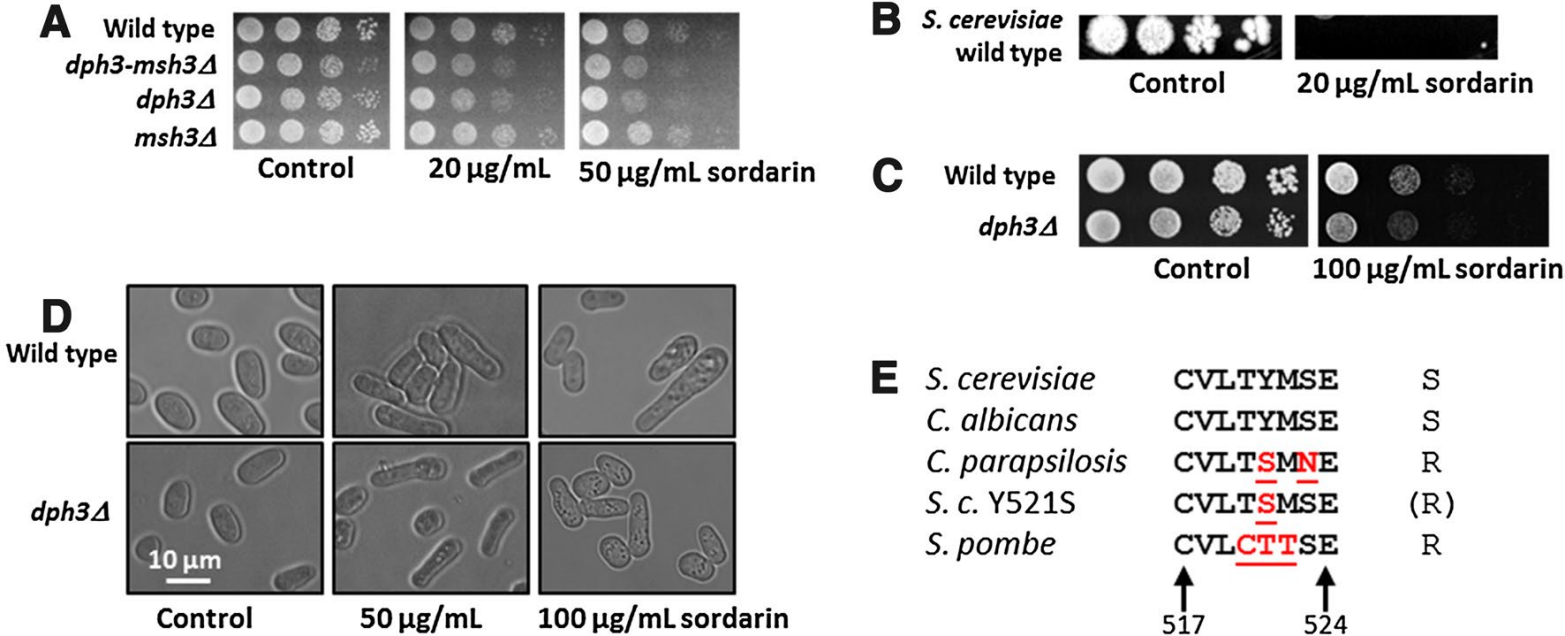
*S. pombe* is resistant to sordarin. **a** *dph3Δ* mutants were more sensitive to sordarin than wild type. Deletion of *dph3*, either alone or in combination with *msh3Δ*, caused sordarin sensitivity when compared to wild type, while the *msh3Δ* single mutant was not more sensitive than wild type. The *msh3* deletion strain used was *msh3::loxP*-*ura4*-*loxM*. An *msh3::kanMX* disruption strain was tested in an independent experiment, which also behaved like wild type (Supplemental Fig. S1). **b** An *S. cerevisiae* wild type strain was extremely sensitive to sordarin. **c** *dph3Δ* was sensitive to a high dose of sordarin when compared to wild type. **d** Sordarin treated *S. pombe* cells were elongated. Wild type and *dph3Δ* mutant cells, taken from plates with the indicated amounts of sordarin and from the control plate, were analysed by microscopy. **e** The sordarin specificity region of eEF2′s of various yeasts as specified by Shastry et al. (2001). Numbers below the alignment indicate the position of the region within the two identical *S. pombe* eEF2 proteins encoded by *eft201* and *eft202*. Amino acid residues that differ from the *S. cerevisiae* sequence are underlined. *S. c.* Y521S is a mutated form of *S. cerevisiae* eEF2 (Shastry et al. 2001). *S* and *R* to the right of the sequences indicate sordarin sensitivity and resistance, respectively. (*R*) indicates intermediate resistance

Parallel to the experiment with the *ATGmut* strains, we tested the effect of sordarin on *S. pombe* strains. We tested the *msh3::kanMX* strain, which was sensitive to HU and MMS (Fig. 1d), the *msh3::loxP*-*ura4*-*loxM* strain, which was not sensitive to HU or MMS (Fig. 2b), the *dph3*–*msh3::ura4* double deletion strain, and the *dph3::loxP*-*ura4*-*loxM* strain and compared them with wild type. Our original expectation was that an impaired *dph3* function causes resistance to sordarin as it has been described for mutations in *S. cerevisiae DPH* genes (Bär et al. 2008; Uthman et al. 2013). However, we learned that *S. pombe* wild type cells were highly resistant to 50 μg/mL sordarin (Fig. 3a), whilst an *S. cerevisiae* wild type strain was extremely sensitive, even at a low concentration of 20 μg/mL (Fig. 3b), consistent with previous results (Shastry et al. 2001; Bär et al. 2008; Uthman et al. 2013). At these concentrations, *dph3Δ* was slightly more sensitive than wild type, while deletions of *msh3* had no effect and the double mutant *dph3*–*msh3Δ* behaved like the *dph3Δ* single mutant (Fig. 3a). When testing sensitivity to high doses of sordarin, it became evident that *dph3Δ* was more sensitive than wild type, whereas the *msh3* deletions were not (Fig. 3c; Supplemental Fig. S1). We further observed that sordarin treated cells were elongated when compared to untreated cells, without any obvious difference between wild type and *dph3Δ* (Fig. 3d).

The specificity region of sordarin in eEF2 of *S. cerevisiae* has been mutagenized and tested for sordarin sensitivity (Shastry et al. 2001). When comparing the amino acid residues of this region in *S. cerevisiae* and *Candida albicans*, yeast species that are highly sensitive to sordarin, with those of *Candida parapsilosis*, and an *S. cerevisiae* eEF2-Y512S mutant, which are sordarin resistant, it is apparent that the presence of at least a tyrosine within this region confers sensitivity (Fig. 3e). In this regard, the *S. pombe* eEF2 proteins clearly differ from those of *S. cerevisiae* and *C. albicans*, and are more similar to the eEF2′s of resistant yeast strains. We conclude that *S. pombe* is largely resistant to sordarin due to the absence of crucial amino acids in the specificity region of eEF2. On the other hand, a mutated *dph3Δ* caused sordarin sensitivity, in contrast to the extreme resistance of the *dph3Δ* mutant of *S. cerevisiae* (Bär et al. 2008; Uthman et al. 2013).

### *msh3::kanMX* did not cause reduced *dph3* expression

**Fig. 4:**
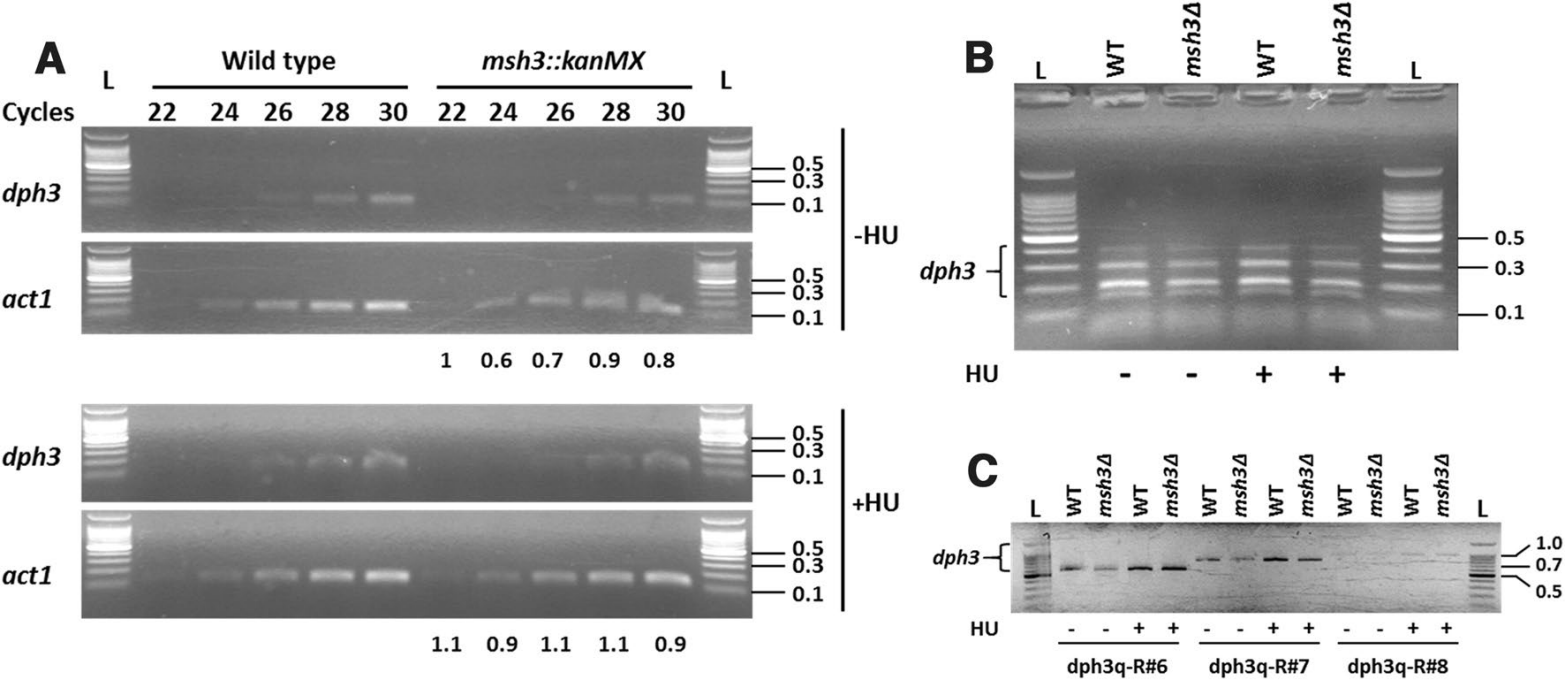
Transcriptional levels and the mRNA size of *dph3* were not altered in *msh3::kanMX* cells. **a** Amplification of *dph3* cDNA derived from reverse-transcribed mRNA with the indicated numbers of PCR cycles. Wild type and *msh3::kanMX* cells were either left untreated or were treated for 4 h with 20 mM HU before RNA isolation. Primers were dph3q-F and dph3q-R. PCR on *act1* cDNA served as a control. Numbers below the bottom gel reflect ratios of *dph3*-specific band intensities between *msh3::kanMX* and wild type after subtracting background and normalising to respective *act1* bands. **b**, **c** The size of *dph3* mRNA was not altered in the *msh3Δ* (*msh3::kanMX*) strain. PCR on cDNA derived from reverse-transcribed mRNA included 30 cycles. **b** Reactions contained dph3q-F and seven reverse primers (dph3q-R#2 to dph3q-R#8). Only the four smallest PCR fragments (approximately 140, 190, 300 and 400 bp) were detectable. **c** PCR for amplification of DNA fragments not detected in the multi-primer reactions shown in (**b**). The reverse primers used are indicated below the gel. Sizes of DNA fragments from *left* to *right* were approximately 600 bp, 830 bp and 1 kb. *Numbers* on the *right* indicate sizes of DNA fragments of a 100-bp DNA ladder (labelled with L) in kb

Since cassette integrations at the *msh3* locus interfered with *dph3* function, we were interested to know whether mRNA levels of *dph3* were affected in the *msh3::kanMX* mutant. mRNA levels of wild type and *msh3::kanMX* strains, either untreated or treated with HU, were determined by semi-quantitative PCR using primers dph3q-F and dph3q-R (Fig. 4a; Supplemental Fig. S2). The forward primer dph3q-F spans intron I, and thus prevents amplification of genomic DNA or unspliced pre-mRNA. We could not find major differences in *dph3* expression between wild type and the *msh3::kanMX* mutant. As a control, we performed PCR on genomic DNA with the same primers. No bands were detected (data not shown), demonstrating that the bands produced with the reverse-transcribed samples reflect amplification of cDNA derived from mRNA. In addition, PCR on a cDNA sample of a *dph3::loxP*-*ura4*-*loxM* strain yielded no bands (Supplemental Fig. S3), demonstrating that the amplified DNA was *dph3* specific. We then used dph3q-F and seven reverse primers for PCR that prime at different positions, covering most of the *dph3* mRNA. We could not detect any relevant differences between wild type and the *msh3::kanMX* mutant (Fig. 4b, c). Thus, altered *dph3* transcription in *msh3::kanMX* cells occurred in a way not detectable with the methods applied. Although our results were negative, we could not come up with alternative explanations other than that transcriptional interference is responsible for the observed drug sensitivity.

### Integration of the *ura4* marker at the *dph3* locus did not affect the Msh3 function in mating-type switching

**Fig. 5:**
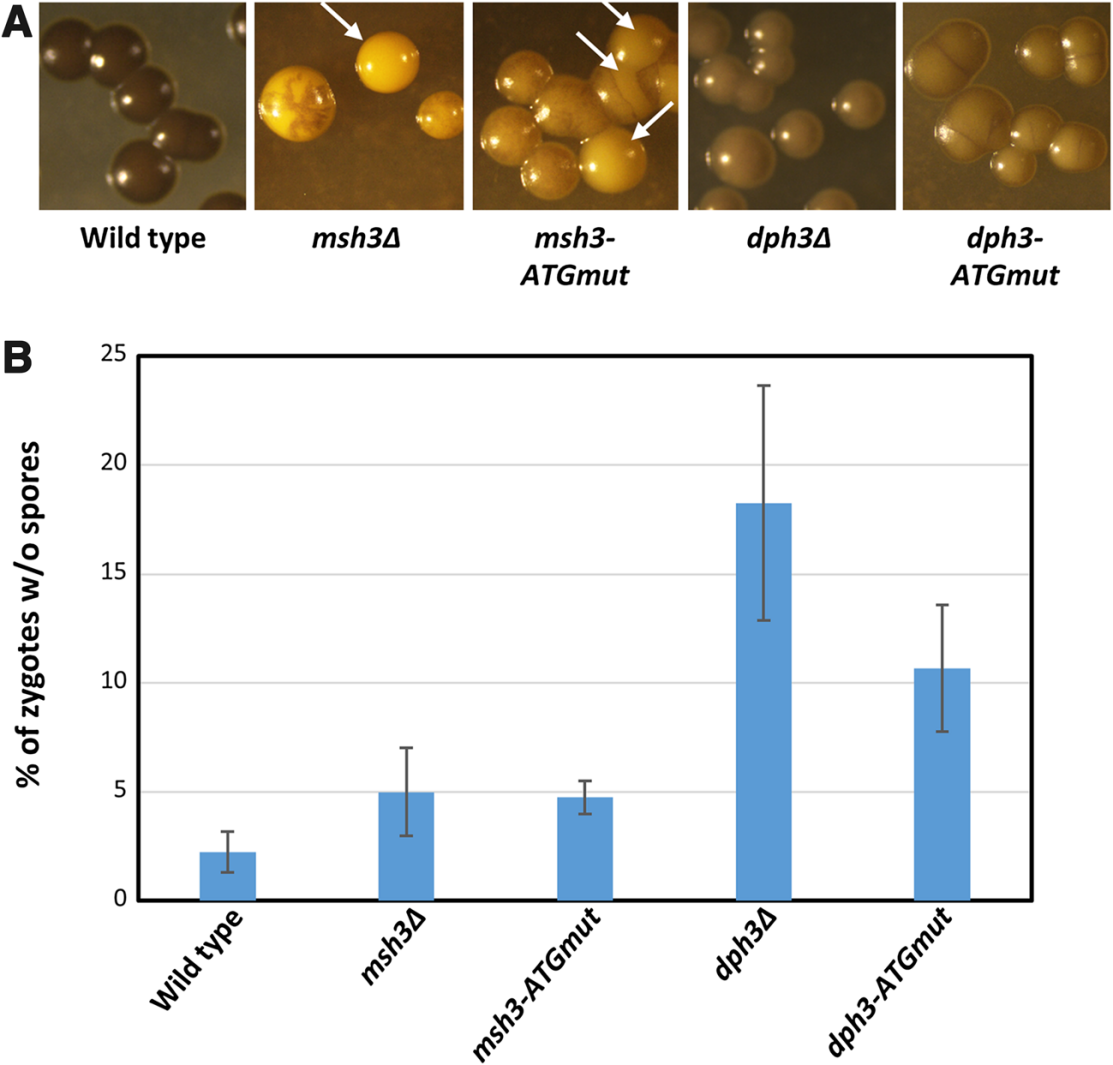
The *dph3* mutants were not affected in mating-type switching, but showed reduced efficiency to undergo meiosis. **a** Mating-type switching. The indicated *h* ^*90*^ strains were streaked on MEA, grown for 4 days and subsequently stained with iodine vapour. Arrows denote iodine-negative colonies containing heterothallic cells of *msh3Δ* and *msh3*-*ATGmut* strains. The other colonies of the *msh3* mutants were mottled. Iodine-negative and mottled colonies were not observed in the *dph3* mutants. **b** *h* ^*90*^ *dph3* mutants frequently produced zygotes without spores. Shown are the means of six independent experiments with standard deviations. Values of *dph3* mutants were significantly different to wild type (*dph3Δ*: *p* = 3 × 10^−5^; *dph3*-*ATGmut*: *p* = 5.1 × 10^−5^), to *msh3Δ* (*dph3Δ*: *p* = 2.1 × 10^−4^; *dph3*-*ATGmut*: *p* = 2.9 × 10^−3^) and to *msh3*-*ATGmut* (*dph3Δ*: *p* = 1.2 × 10^−4^; *dph3*-*ATGmut*: *p* = 7 × 10^−4^). p-values were calculated by a two-tailed Student’s *T* test

Since *msh3* disruptions interfered with Dph3 function, we asked whether also the opposite occurred, i.e. whether the *dph3::loxP*-*ura4*-*loxM* cassette integration affected Msh3 function. For this we tested the mating-type switching competence of an *h* ^*90*^ *dph3::loxP*-*ura4*-*loxM* mutant. Msh3, formerly known as Swi4, is involved in mating-type switching (Fleck et al. 1990; Egel 2005). Homothallic *h* ^*90*^ *msh3* mutants form so-called mottled colonies and frequently produce duplications in the mating-type region, leading to heterothallic *h* ^+^ cells (Egel et al. 1984; Fleck et al. 1990, 1992). Wild-type colonies turned homogeneously brown after iodine staining (Fig. 5a) (for explanation of the mating-type switching assay see “Materials and methods”). The *h* ^*90*^ *msh3::loxP*-*ura4*-*loxM* and *msh3*-*ATGmut* strains formed mottled and iodine-negative (approximately 60%) colonies, indicative for a switching defect. The *h* ^*90*^ *dph3::loxP*-*ura4*-*loxM* and *h* ^*90*^ *dph3*-*ATGmut* mutants formed iodine positive colonies, although less homogeneously stained than wild type (Fig. 5a). The *dph3* mutants mainly produced four-spored asci, but unlike wild type, also had many empty zygotes without spores (Fig. 5b). Importantly, mottled and iodine-negative colonies with heterothallic cells were not observed among thousands of colonies. Thus, mating-type switching was not affected, indicating that Msh3 is functional in *dph3Δ* background.

## Discussion

We have discovered that replacement of the *S. pombe msh3* gene by *kanMX* or *hphMX* interfered with the function of the adjacent *dph3* gene. Such *msh3* gene disruptions rendered cells sensitive to HU and MMS. The *dph3* and *msh3* genes are closely linked and transcribed in opposite directions from divergent and likely overlapping promoters or by one bidirectional promoter (Li et al. 2015) (Fig. 1a). It is unlikely that the cassettes replacing *msh3* delete part of the 5′ UTR or vital promoter elements of *dph3*, since *loxP*-*ura4*-*loxM*, which replaced essentially the same DNA portion as *kanMX* and *hphMX*, was not sensitive to HU or MMS (Fig. 2b). The 34-nucleotide long sequence between the two supposed transcription start sites contains 5′-TTTATAT-3′, which can be used as TATA box from both directions. Interestingly, many DNA repair genes in the human genome, including *MSH3*, share a short promoter region with a flanking gene (Adachi and Lieber 2002). In the case of *MSH3*, the flanking gene is *DHFR*, which is involved in the synthesis of nucleic acid precursors (Fujii and Shimada 1989). The DHFR enzyme is a target of the chemotherapeutic agent methotrexate (Goodsell 1999). A methotrexate resistant cell line has been shown to have simultaneously up-regulated *DHFR* and *MSH3*, which led to imbalanced MMR complexes and thereby reduced the efficiency of repair of base–base mismatches (Drummond et al. 1997). It is, therefore, conceivable that expression of *dph3* and *msh3* in *S. pombe* is also co-regulated.

The cassettes *kanMX* and *hphMX* contain the strong *TEF* promoter of the *Ashbya gossypii TEF* gene encoding translation elongation factor 1 alpha (Steiner and Phillippsen 1994; Bähler et al. 1998; Hentges et al. 2005). In both cases, the *TEF* promoter is in close proximity to the *dph3* promoter. In the *msh3::loxP*-*ura4*-*loxM* disruption, *ura4* is expressed by its own promoter, which is also located next to *dph3*. However, this construction did not cause HU or MMS sensitivity (Fig. 2b). Approximately, five mRNA molecules of the *ura4* gene are present in vegetative growing cells (Marguerat et al. 2012). It is, therefore, conceivable that the presence of the strong *TEF* promoter, but not of the weaker *ura4* promoter, interfered with transcription of the *dph3* gene. Transcriptional interference of flanking genes can occur naturally and often reflects a regulatory role in gene expression. Transcriptional interference can also be caused by integration of foreign DNA, e.g. by transposons and viruses, or artificially by genetic manipulations (Shearwin et al. 2005). For example, cassette integrations at the mouse *MRF4* gene interfered with the function of the nearby *Myf5* gene when cassettes were transcribed towards *Myf5*, but not when transcribed in the opposite direction (Olson et al. 1996). When testing *dph3* mRNA levels and size, we could not detect any obvious difference between wild type and the *msh3::kanMX* mutant (Fig. 4). Thus, although transcriptional interference by cassette integration is the likely cause for the observed phenotypes, we were not able to support this by experimental evidence. A study in *S. cerevisiae* revealed that it is a common feature that cassette integrations affect the function of a neighbouring gene (Ben-Shitrit et al. 2012). Among nine selected genes, only five showed a significantly altered mRNA level. Thus, transcriptional interference of the remaining four genes, as for the *S. pombe dph3* gene, likely occurred by subtle changes in expression or by structural changes of the mRNAs, which were not detectable with the methods applied.

We noticed that colonies of the *h* ^*90*^ *dph3::loxP*-*ura4*-*loxM* and *h* ^*90*^ *dph3*-*ATGmut* strains were less homogenously stained by iodine vapour than *h* ^*90*^ wild-type colonies (Fig. 5a). Both *dph3* mutants contained many zygotes without spores (Fig. 5b). On the other hand and in contrast to *msh3* mutants, mottled and iodine negative (heterothallic) colonies were not formed. Thus, replacement of *dph3* by the *ura4* marker did not disrupt the Msh3 function in mating-type switching. The phenotype of reduced sporulation of *dph3* mutants is unrelated to Msh3 functions, as *dph3*-*ATGmut* does not have an integrated cassette that may interfere with *msh3* expression.

Mutated *dph3* (*kti11*), like the other *dph* mutated genes in *S. cerevisiae* causes resistance to diphtheria toxin and sordarin due to the lack of the diphthamide modification of eEF2 (Schaffrath et al. 2014). *dph3* mutants are also resistant to the killer toxin of *Kluyveromyces lactis*, like Elongator (*elp*) mutants and unlike the other *dph* mutants (Fichtner and Schaffrath 2002; Bär et al. 2008). In this case, resistance was due to the absence of a mcm^5^U_34_ modification of tRNAs, which the killer toxin requires for cleavage of the tRNAs. We have found that *S. pombe* wild type cells were highly resistant to the fungicide sordarin, but became sensitive when *dph3* was deleted (Fig. 3). This is in contrast to *S. cerevisiae*, where wild type cells are extremely sensitive and deletions of any of the *DPH* genes confer resistance (Bär et al. 2008; Uthman et al. 2013). The different impact of sordarin on wild type cells of both yeasts is likely due to differences in the sordarin specificity region of the eEF2 proteins as defined by Shastry et al. (2001) (Fig. 3e). The reason why *S. pombe dph3Δ* is sensitive to sordarin is currently unknown and should be investigated in future studies. One possibility is that lack or reduction of tRNA modifications exacerbate the effect of sordarin, which stalls eEF2 on ribosomes and thereby blocks ribosomal translocation during translation of mRNAs (Justice et al. 1998; Domínguez et al. 1999). However, this would mean that high sordarin concentrations can inhibit eEF2 without the diphthamide modification. We further found that wild type and *dph3Δ* mutant cells were elongated when treated with sordarin (Fig. 3d), indicating a defect in cell cycle progression, where cells were able to grow but had problems to divide.

In *S. pombe elp3* mutants, protein levels of Cdr2, Atf1, and Pcr1 are low (Bauer et al. 2012; Fernández-Vázquez et al. 2013). The Cdr2 kinase positively regulates the G2/M transition of the cell cycle, while Atf1-Pcr1 controls cellular stress responses on the transcriptional level. *elp3* mutants were sensitive to hydrogen peroxide, rapamycin and to incubation at 36 °C. These phenotypes could be rescued by elevated levels of ${\text{tRNA}}_{\text{UUU}}^{\text{Lys}}$ expressed from multi-copy plasmids (Bauer et al. 2012; Fernández-Vázquez et al. 2013). This demonstrates that the inability to modify this tRNA species in *elp3* cells caused the observed phenotypes. Lack of the mcm^5^∪_34_ modification leads to inefficient decoding of AAA, CAA and GAA codons. Consequently, genes with a high number of AAA codons (vs. AAG) for lysine have low protein levels in *elp3* mutants (Bauer et al. 2012; Fernández-Vázquez et al. 2013). Indeed, when the AAA codons of *cdr2* or *atf1* were changed to AAG, protein levels returned to the wild-type level (Bauer et al. 2012; Fernández-Vázquez et al. 2013). Since Dph3 acts together with Elp3 in tRNA modifications, it is plausible that lack of such modifications affect synthesis of specific proteins required for a proper response of the cells to HU and/or MMS treatment.

We have found in the present study that *S. pombe dph3* mutations caused sensitivity to the cytotoxic agents HU and MMS. Thus, Dph3 acts in response to DNA damage and replication stress, likely by tRNA and/or eEF2 modifications, which ensure efficient translation of proteins required for such processes. To our knowledge, a function for Dph3 in response to DNA damaging drugs has not been described yet. An *S. cerevisiae elp3* mutant showed some sensitivity to HU and MMS, which was further increased in double mutants additionally defective in the histone acetyltransferase Rtt109, the histone chaperones Asf1 and Cac1 or mutated in PCNA (Li et al. 2009). Future work should address how other *S. pombe* mutants of the Dph pathway and of the Elongator complex react to DNA damaging agents and which phenotypes are due to impaired tRNA modifications or loss of the eEF2 diphthamide modification. Such studies might give valuable information about the choice of drugs in cancer chemotherapy in relation to the status of the Dph and Elongator pathways in tumour cells. It has been found that approximately 80% of ovarian cancers have *DPH1* gene deletions and that ELP3 is up-regulated in breast cancer cells, which in turn leads to increased levels of the oncoprotein DEK (Chen and Behringer 2004, 2005; Kong et al. 2011; Delaunay et al. 2016). *DPH3* promoter mutations are present in up to 16% of melanomas and silencing of *DPH3* in mouse melanoma cells impairs metastasis (Wang et al. 2012; Denisova et al. 2015; Fredriksson et al. 2014). Thus, a combination of drug treatment with targeting specific factors of the modification pathways appears to be a sensible approach to sensitize rapidly growing cancer cells. Finally, it is important to better understand the antifungal mode of action of drugs such as sordarin for selective and efficient killing of pathogenic fungal strains, which is especially crucial for immunocompromised patients, for example for those suffering from AIDS or cancer.

## Materials and methods

### Yeast media, genetic methods and strains

Yeast media were YEA (yeast extract agar), YEL (yeast extract liquid), MEA (malt extract agar) and MMA (minimal medium agar) as described (Gutz et al. 1974) with the following modified composition of MMA: 0.17% yeast nitrogen base without amino acids/ammonium sulphate/thiamine, 0.5% ammonium sulphate, 1% glucose, 1.8% granulated agar (Formedium). The supplements adenine, histidine, leucine and uracil, were added at concentrations of 100 mg/L to complex media and MEA, and to MMA where required. G418 (100 mg/L), hygromycin B (200 mg/L) and 5-fluoroorotic acid (1 g/L) were added to YEA after autoclaving. Drug containing plates were prepared 2 days before use and until then stored at room temperature. Strains were grown overnight to stationary phase and cell titres were determined with a haemocytometer. Cultures were diluted to 10^7^ cells/mL in sterile H_2_O and three consecutive 1:10 dilutions were prepared. Ten microliter of all four dilutions were spotted on drug containing plates and on control plates without drugs. Images were taken with a Gel Doc 2000 system (Bio-Rad) after 3 days of incubation at 30 °C. Drugs used were HU (Formedium), MMS (ACROS Organics) and sordarin (Santa Cruz). Spot tests were carried out at least twice, with the exception of the experiments including the plates with 100–150 μg/mL sordarin. To determine spore formation efficiency 10 μL of 1:10 dilutions in H_2_O of stationary phase cultures were spotted on MEA and incubated for 2 days at 30 °C. Means and standard deviations were calculated from six independent experiments, where 200 units (asci and zygotes) were counted for each of them. *S. pombe* strains are listed in Supplemental Table S1. The *S. cerevisiae* strain RCY2459 (*MATa ho::LYS2 ura3 lys2 leu2::hisG* in SK1 background) was a kind gift of Rita Cha (NWCR Institute, Bangor University).

### Construction of *msh3* and *dph3* gene disruption strains

FA10 (*h* ^−^ *msh3::kanMX*) was obtained by transformation of the 972 wild type strain (*h* ^−^) with a PCR product obtained with primers D3kf 5′-TTTTTAAAACGTCCGCCTATATTCATTTAGAAACTATAAATGCGTTACAAATCTATGTTGAGCAAATTTGATTGTACAGTTTTTATTTTCGCTTTATTACCGCCAGCTGAAGCTTCGTAC-3′ and Dk3r 5′-AGAACTTAAAACTGATGTATAGTTGCTTAAATTTATTTATACAGTAAAATCTTATGTGTTGAGTATCCTAATAAAGTAGTTAATTCAGTAATGCTCTCTCGGCCACTAGTGGATCTGATA-3′ using plasmid pFA6a-kanMX (Bähler et al. 1998) as template.

KK11 (*h* ^−^ *msh3::hphMX ura4*-*D18*) was constructed by transformation of OL2137 (*h* ^−^ *ura4*-*D18*) with a PCR fragment obtained with primers D3kf and Dk3r (see above) and pFA6a-hphMX6 (Hentges et al. 2005) as template.

KK83 (*smt*-*0 msh3::loxP*-*ura4*-*loxM leu1*-*32 ura4*-*D18*) derived from transformation of EH238 (*smt*-*0 leu1*-*32 ura4*-*D18*) with a PCR fragment obtained with primers msh3_pAW1_For 5′-TTTTTAAAACGTCCGCCTATATTCATTTAGAAACTATAAATGCGTTACAAATCTATGTTGAGCAAATTTGATTGTACAGTTTTTATTTTCGCTTTATTACCGGATCCCCGGGTTAATTAA-3′ and msh3_pAW1_Rev 5′-AGAACTTAAAACTGATGTATAGTTGCTTAAATTTATTTATACAGTAAAATCTTATGTGTTGAGTATCCTAATAAAGTAGTTAATTCAGTAATGCTCTCTCGAATTCGAGCTCGTTTAAAC-3′ using pAW1 (Watson et al. 2008) as template.

DE4 (*h* ^−^ *dph3::loxP*-*ura4*-*loxM ura4*-*D18*) is a transformant of OL2137 with a PCR fragment obtained with primers dph3_pAW1_For2 5′-CTTAGCTTGTAGTTTTCATTATGGGCGGTTCCCACATATAAAACAAATTTTTGGTGGAGTGGCCACGCACCTTCTGCCAGTAGTGCATTGAAGCGGCAAAAGCTTAGCTACAAATCCCAC-3′ and dph3_pAW1_Rev2 5′-TATCACGATGTAAAGAGTAGCCCTCCTATCCTTCGTAATTTCAAGACATTTTGAGAATAAATAGAAGTAAAAAACCAAATAAGAAATTATAGGGAAAAAAGCTTGTGATATTGACGAAAC-3′ and pAW1 as template.

### Cloning of *msh3* into pAW8

Plasmid pAW8-msh3 was constructed by in vitro Cre recombination between the *lox* sites of PCR fragments containing the entire *msh3* gene and the *lox* sites of pAW8-ccdB (a kind gift of Edgar Hartsuiker; NWCR Institute, Bangor University), a derivative of pAW8 (Watson et al. 2008) containing the *ccdB* gene for counter-selection. Primers for amplification of *msh3* were msh3_pAW8_For 5′-GCATGGCGGCCGCATAACTTCGTATAGCATACATTATACGAAGTTATATGCACCACCACCACCACCACGGAGGAGGAAGAGGAATGAGTTATAACAT-3′ and msh3_pAW8_Rev 5′-TTCGCGCGGCCGCATAACTTCGTATATAATACCATATACGAAGTTATTCAGAGCTCTTCGAAAGCGGTAAG-3′. Nucleotides underlined in msh3_pAW8_For encode a (His)_6_ tag followed by three glycine-encoding codons, added between the natural ATG start codon and the second codon of *msh3*. Nucleotides underlined in msh3_pAW8_Rev 5′ changed the two last codons of *msh3* from GAA ATC (encoding the amino acids glutamic acid and isoleucine) to GAG CTC (encoding glutamic acid and leucine), thereby introducing a *Sac*I restriction site, allowing further manipulation if desired.

### Construction of *msh3* and *dph3* strains with mutated ATG start codons

Strain DE5 (*h* ^−^ *dph3*-*ATGmut ura4*-*D18*) was constructed by transformation of DE4 with a PCR fragment obtained with primers dph3_ATGmut_For 5′-TAGCTTGTAGTTTTCATTATGGGCGGTTCCCACATATAAAACAAATTTTTGGTGGAGTGGCCACGCACCTTCTGCCAGTAGTGCATTGAAGCGGCAAA-TGATCATTTTACGACGAAATCG-3′ and dph3_Rev 5′-TCACGATGTAAAGAGTAGCCCTCCTATCCTTCGTAATTTCAAGACATTTTGAGAATAAATAGAAGTAAAAAACCAAATAAGAAATTATAGGGAAAAATTATGCTGCAATGATTATAGGTG-3′ and as template genomic DNA of strain RO144 (*smt*-*0*). “-”in primer dph3_ATGmut_For indicates deletion of an A of the wild type sequence, which causes an in frame TGA stop codon instead of the ATG start codon. By this procedure, the *lox* sites flanking *dph3* in DE4 were replaced in DE5. Sequencing confirmed the correct mutation in DE5 and an additional T155A point mutation, which is in intron II of the *dph3* gene.

DE7 (*smt*-*0 msh3*-*ATGmut leu1*-*32 ura4*-*D18*) originated from the transformation of KK83 with a PCR fragment obtained from a mutagenized pAW8-msh3 plasmid as template. This plasmid contains the *msh3* gene, with the 31st codon mutagenized from ATG to ATC by site directed mutagenesis with a QuikChange lightning site-directed mutagenesis kit (Agilent Technologies) using primers msh3_M31I_S 5′-GGAGCAATATCAGAAGATATCGTTGCCCTCAGTGGTCCAG-3′ and msh3_M31I_AS 5′-CTGGACCACTGAGGGCAACGATATCTTCTGATATTGCTCC-3′. Nucleotides deviating from the wild-type sequence are underlined. Primers for amplifying the 5′ part of the open reading frame of *msh3* with ATG mutations at codons 1 and 4 were msh3-ATGmut_For2 5′-TCCGCCTATATTCATTTAGAAACTATAAATGCGTTACAAATCTATGTTGAGCAAATTTGATTGTACAGTTTTTATTTTCGCTTTATTACTCGAGAGGATAGAGTTATAACATTACTCATG-3′ and msh3_Rev2 5′-CTTAAAACTGATGTATAGTTGCTTAAATTTATTTATACAGTAAAATCTTATGTGTTGAGTATCCTAATAAAGTAGTTAATTCAGTAATGCTCTCTCTCAGATTTCTTCGAAAGCGGTAAG-3′. Primer msh3-ATGmut_For2 contained base substitutions (underlined), which changed the ATG start codon of *msh3* to TCG and the fourth codon from ATG to TAG. After integration into the genome, the mutated *msh3* gene was amplified by PCR and sequencing confirmed the three desired ATG mutations. In addition, we found A555G, C696T and T1112C mutations. The primers used for PCR to construct an ATG mutated *msh3* strain were designed in such a way that the (His)_6_ tag, the glycine linker and the *lox* sites of plasmid pAW8-msh3-M31I were not present in the genome of the resulting DE7 strain.

### *dph3* expression

10 mL cultures were grown in YEL at 30 °C to a density of approximately 10^6^ cells/mL and 5 mL were taken out, pelleted, frozen in liquid nitrogen and stored at −80 °C. 20 mM HU was added to the remaining cultures, which were further incubated for 4 h. Cells were pelleted, frozen in liquid nitrogen and stored at −80 °C. RNA was isolated from untreated and HU-treated cells using a MasterPure Yeast RNA Purification kit (epicentre) including DNase I treatment. 2 µg of RNA were reverse transcribed with oligo (dT)_18_ primers using a Tetro cDNA Synthesis kit (Bioline). The resulting cDNA was subjected to 22–35 cycles of 30 s at 94 °C, 30 s at 57 °C and 30 s at 72 °C and a final extension for 10 min at 72 °C. For amplification of *dph3* cDNA the forward primer dph3q-F 5′-AGATTTCACGTTTGACGCCG-3′ (spanning intron I of *dph3,* and thus prevents amplification of contaminating genomic DNA) and the reverse primer dph3q-R 5′-CTTGGGCAACGAGCAACATC-3′ was used. In a pilot experiment (Supplemental Fig. S3), we used primers dph3_cPCR_For2 5′-CTATGATGAAGATGAATTCATGGAAGTTG-3′ and dph3_cPCR_Rev2 5′-GAACAACAAACGTACTATGCCAATACAAAACC-3′. Other reverse primers for amplification of *dph3* cDNAs of various sizes were dph3q-R#2 5′-TCACCCGGACAATCAAGCTG-3′, dph3q-R#3 5′-AGGTGCTGTAGAAGCATCGT-3′, dph3q-R#4 5′-GTAGCCCTCCTATCCTTCGT-3′, dph3q-R#5 5′-TGGAAAAGTCGTACGCTCAA-3′, dph3q-R#6 5′-AGGTGGGCTTTTAGTTTCGAGT-3′, dph3q-R#7 5′-ACGTTGAGCACAAGTACGAA-3′ and dph3q-R#8 5′-CCACTGTGGTATGTCGCACT-3′. Primers for amplification of *act1* cDNA were act1-For 5′-AAGTACCCCATTGAGCACGG-3′ and act1-Rev 5′-CAGTCAACAAGCAAGGGTGC-3′. PCR products were separated on 1.5% agarose gels in Tris–Borate-EDTA buffer and images taken with a Gel Doc 2000 system (Bio-Rad). Band intensities of PCR products were quantified with the ImageJ software (NIH). *dph3*-specific bands were normalised to blanks to subtract background and to *act1* cDNA.

### Mating-type switching assay

Iodine vapour stains spores but not vegetative *S. pombe* cells (Forsburg and Rhind 2006). On sporulation medium, colonies of a switching competent *h* ^*90*^ wild-type strain turn homogeneously brown when stained with iodine, while colonies of non-sporulating heterothallic strains are yellow. *h* ^*90*^ *msh3* mutants form mottled colonies and frequently segregate iodine-negative *h* ^+^ colonies due to duplications in the mating-type region (Egel et al. 1984; Fleck et al. 1990). Images of iodine-stained colonies were taken with a LEICA MZ10F microscope using LEICA Application Suite V3 software. To determine the frequency of heterothallic colonies, three *h* ^*90*^ colonies of each strain were streaked on MEA and grown for 3 days at 30 °C. From each original *h* ^*90*^ colony, approximately 300–500 colonies were inspected after iodine staining.

## Electronic supplementary material

Below is the link to the electronic supplementary material.

Supplementary material 1 (PDF 426 kb)

Supplementary material 2 (DOCX 15 kb)
