# Supplementary material for: A mutated dph3 gene causes sensitivity of Schizosaccharomyces pombe cells to cytotoxic agents
Source: Curr Genet. 2017 May 29;63(6):1081–91. doi: 10.1007/s00294-017-0711-x (PMC5668335; doi:10.1007/s00294-017-0711-x)
Supplement: Supplementary file 1 — Supplementary material 1 (PDF 426 kb) [file 294_2017_711_MOESM1_ESM.pdf]

Current Genetics

A mutated *dph3* gene and nearby cassette integrations cause sensitivity of *Schizosaccharomyces pombe* cells to cytotoxic agents

Desirée Villahermosa[1] Karen Knapp[1,2] Oliver Fleck[1,*]

* Corresponding author: Oliver Fleck

E-mail: o.fleck@bangor.ac.uk

[1] North West Cancer Research Institute, Bangor University, Bangor LL57 2UW, United Kingdom

[2] Present Address: Department of Pathology, Dunedin School of Medicine, University of Otago, Dunedin 9054, New Zealand.

Supplemental Fig S1

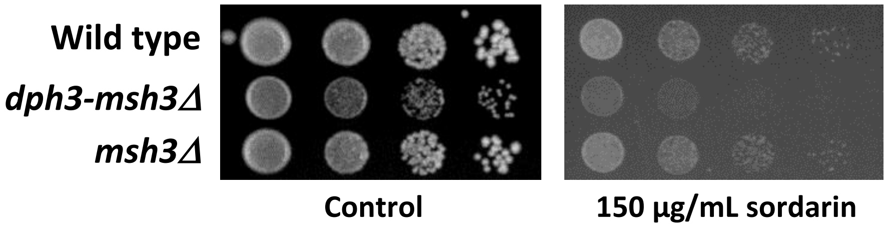

Fig. S1 Test for sordarin sensitivity of *dph3-msh3Δ* and *msh3Δ*. The latter strain was FA10 (*msh3::kanMX*).

Supplemental Fig S2

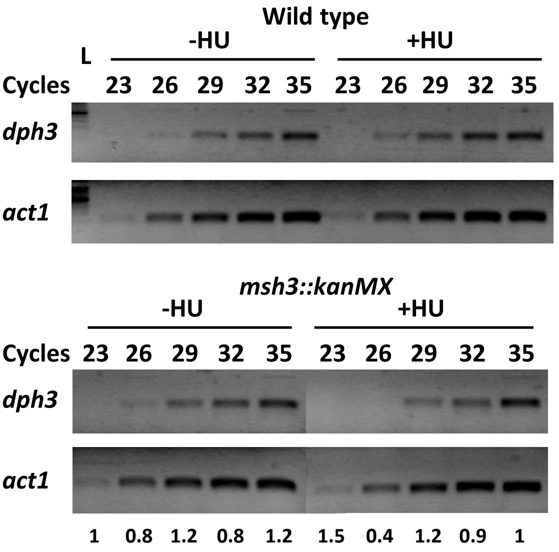

Fig. S2 Repeated experiment for measuring expression of *dph3*. PCR with the indicated number of cycles was performed on cDNA derived from reverse-transcribed mRNA of untreated and HU treated wild type and *msh3::kanMX* strains. Numbers below the gels are the ratios of intensities of *dph3*-specific bands in *msh3* versus wild type background after normalised to the *act1* control.

Supplemental Fig S3

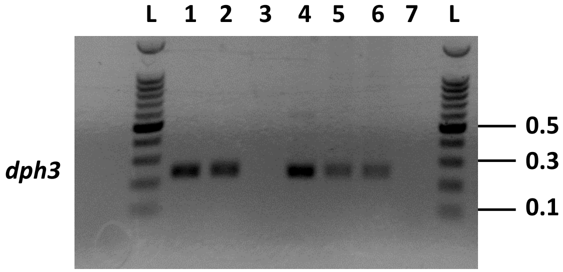

Fig. S3 *dph3* expression of various *S. pombe* strains. PCR was performed for 30 cycles on cDNA derived from reverse-transcribed mRNA of untreated cells using primers dph3_cPCR_For2 and dph3_Rev2. The resulting DNA fragment was 238 bp long. Lane 1: RO144 (wild type); lane 2: DE7 (*msh3-ATGmut*); lane 3: DE4 (*dph3-loxP-ura4-loxM*); lane 4: KK11 (*msh3::hphMX*); lane 5: KK83 (*msh3::loxP-ura4-loxM*); lane 6: FA10 (*msh3::kanMX*); lane 7: KK11 without reverse transcriptase. Numbers on the right indicate sizes of DNA fragments of a 100-bp DNA ladder (labelled with L) in kb.
