# Supplementary material for: A mutated dph3 gene causes sensitivity of Schizosaccharomyces pombe cells to cytotoxic agents
Source: Curr Genet. 2017 May 29;63(6):1081–91. doi: 10.1007/s00294-017-0711-x (PMC5668335; doi:10.1007/s00294-017-0711-x)
Supplement: Supplementary file 2 — Supplementary material 2 (DOCX 15 kb) [file 294_2017_711_MOESM2_ESM.docx]

**Supplemental Table S1** *S. pombe* strains

| Name | Genotype | Origin |
| --- | --- | --- |
| 972 | *h^-^* | Urs Leupold, Bern |
| DE4 | *h^-^ dph3::loxP-ura4-loxM ura4-D18* | This study |
| DE5 | *h^-^ dph3-ATGmut ura4-D18* | This study |
| DE7 | *smt-0 msh3-ATGmut leu1-32 ura4-D18* | This study |
| EH238 | *smt-0 leu1-32 ura4-D18* | Edgar Hartsuiker, Bangor |
| FA10 | *h^-^ msh3::kanMX* | This study |
| KK11 | *h^-^ msh3::hphMX ura4-D18* | This study |
| KK83 | *smt-0 msh3::loxP-ura4-loxM leu1-32 ura4-D18* | This study |
| OL712 | *h^-^ msh6::arg3 arg3-D4* | Derived from CT-1; Tornier et al. 2001 |
| OL1348 | *h^-^ msh2::his3 his3-D1* | Derived from Ru39; Rudolph et al. 1999 |
| OL1394 | *h^90^* | Strain collection |
| OL2091 | *h^-^ mlh1::kanMX* | Derived from OL937; Marti et al. 2003 |
| OL2137 | *h^-^ ura4-D18* | Strain collection |
| OL2307 | *h^90^ msh3::loxP-ura4-loxM leu1-32 ura4-D18* | This study |
| OL2384 | *h^90^ msh3-ATGmut leu1-32 ura4-D18* | This study |
| OL2551 | *h^90^ dph3-ATGmut* | This study |
| OL2565 | *h^90^ dph3::loxP-ura4-loxM ura4-D18* | This study |
| PRS301 | *h^-^ pms1::ura4 ura4-D18* | Schär et al. 1997 |
| RO144 | *smt-0* | Rolf Kraehenbuehl, Bangor |
| Ru106 | *h^+^ dph3-msh3::ura4 ura4-D18* | Derived from LH110, Fleck et al. 1992 |

References

Fleck O, Michael H, Heim L (1992) The *swi4*^+^ gene of *Schizosaccharomyces pombe* encodes a homologue of mismatch repair enzymes. Nucleic Acids Res 20:2271-2278

Mansour AA, Tornier C, Lehmann E, Darmon M, Fleck O (2001) Control of GT repeat stability in *Schizosaccharomyces pombe* by mismatch repair factors. Genetics 158:77-85

Marti TM, Mansour AA, Lehmann E, Fleck O (2003) Different frameshift mutation spectra in non-repetitive DNA of MutSalpha- and MutLalpha-deficient fission yeast cells. DNA Repair (Amst) 2:571-580

Schär P, Baur M, Schneider C, Kohli J. (1997) Mismatch repair in *Schizosaccharomyces pombe* requires the *mutL* homologous gene *pms1*: molecular cloning and functional analysis. Genetics 146:1275-1286

Tornier C, Bessone S, Varlet I, Rudolph C, Darmon M, Fleck O (2001) Requirement for Msh6, but not for Swi4 (Msh3), in Msh2-dependent repair of base-base mismatches and mononucleotide loops in *Schizosaccharomyces pombe*. Genetics 158:65-75
